# Association Between Psoriasis and Subclinical Atherosclerosis: A Meta-Analysis

**DOI:** 10.1097/MD.0000000000003576

**Published:** 2016-05-20

**Authors:** Na Fang, Menglin Jiang, Yu Fan

**Affiliations:** From the Institute of Molecular Biology & Translational Medicine, the Affiliated People's Hospital, Jiangsu University, Zhenjiang, Jiangsu, PR China.

## Abstract

The association between psoriasis and carotid intima-media thickness (CIMT) or impaired flow-mediated dilation (FMD) remains controversial. We aimed to evaluate the extent of subclinical atherosclerosis as measured by CIMT and FMD in patients with psoriasis by conducting a meta-analysis.

A systematic literature search was performed using PubMed, Embase, Cochrane databases, China National Knowledge Infrastructure, and VIP databases up to February 2015. Observational studies investigating CIMT or FMD in patients with psoriasis and controls were eligible. Psoriatic patients and controls were at least age- and sex-matched. Random-effects analysis was used to estimate the weighted mean difference (WMD) and 95% confidence interval (CI) between psoriatic patients and controls.

A total of 20 studies were identified and analyzed. Meta-analysis showed that psoriatic patients had a significantly thicker CIMT (WMD 0.11 mm; 95% CI 0.08–0.15) and lower FMD (WMD −2.79%; −4.14% to −1.43%) than those in controls. Subgroup analysis indicated that psoriatic arthritis appeared to have less impaired FMD (WMD −2.45%) and thinner CIMT (WMD 0.10 mm). Psoriatic patients with mean age >45 years had much thicker CIMT (WMD 0.13 mm). The impaired FMD (WMD −3.99%) seemed more pronounced in psoriatic patients with mean age <45 years.

This meta-analysis suggests that patients with psoriasis are associated with excessive risk of subclinical atherosclerosis. Screening and monitoring CIMT and brachial artery FMD may be recommended to identify a subgroup of psoriatic patients at higher risk for cardiovascular events.

## INTRODUCTION

Psoriasis is a chronic inflammatory skin disease characterized by relapsing thick scaling plaques.^1^ The prevalence ranged from 0.91% to 8.5% in the adult population.^2^ Among psoriatic patients, approximately 6% to 42% of the whites^3^ and 1% to 9% Asian patients^4^ were reported to have psoriatic arthritis. Psoriatic arthritis is defined as inflammatory arthritis associated with psoriasis. Psoriasis not only negatively affects the quality of life, but also increases risk of cardiovascular events^5^ and cardiovascular mortality.^6^ Therefore, early detection of subclinical atherosclerosis in psoriatic patients would help to reduce cardiovascular morbidity and mortality.

Endothelial function^7^ and carotid intima-media thickness (CIMT)^8^ have been suggested to be an important marker of subclinical atherosclerosis. Assessment of flow-mediated dilation (FMD) with high-resolution ultrasound in the brachial artery is a widely used method to evaluate the endothelial function.^9^ CIMT is usually determined by using B-mode ultrasound technique in the common carotid artery. Determination of FMD and CIMT is widely used in clinical practice because of their noninvasive technique. Most studies have shown evidence of subclinical atherosclerosis in psoriatic patients as indicated by increased CIMT^10–23^ or impaired FMD^14,15,17,19,24–26^ than the matched controls. However, conflict findings regarding the relationship between psoriasis and subclinical atherosclerosis risk still exist.^27–30^ These conflicting results might be correlated with the severity or duration of psoriasis and population studied.

This meta-analysis aims to quantitatively estimate the association between psoriasis and subclinical atherosclerosis as measured by CIMT and FMD in patients with psoriasis by conducting a meta-analysis.

## METHODS

### Search Strategy

This study was conducted according to the recommendations of the Meta-Analysis of Observational Studies in Epidemiology.^31^ This meta-analysis was not based on the individual participant data; ethical approval was not applicable. A systematic search of studies published before February 2015 was conducted through PubMed, Embase, Cochrane databases, China National Knowledge Infrastructure, and VIP databases. The following medical subject headings terms were used for the literature search: “psoriasis” OR “psoriatic arthritis” AND “carotid intima-media thickness” OR “carotid atherosclerosis” AND “endothelial function” OR “flow-mediated dilation” AND “subclinical atherosclerosis”. Only fully published articles in peer-reviewed journals were included. The references of retrieved articles were also reviewed to identify any relevant study.

### Inclusion and Exclusion Criteria

Inclusion criteria were: observational studies investigating the relationship between psoriasis with or without psoriasis arthritis and endothelial function (determination by FMD of the brachial artery using ultrasound technique) or mean CIMT; reporting CIMT or FMD as continuous data for patients with psoriasis and controls; psoriatic patients and controls were at least age- and sex-matched. Exclusion criteria were: lack of an eligible control group; evaluating endothelial function except for FMD; studies did not provide CIMT or FMD as mean values and standard deviation (SD) or standard error.

### Data Extraction and Quality Assessment

The following data were extracted from each included study: first author's name, publication year, geographic region, study design, type of psoriasis, psoriasis severity (Psoriasis Area and Severity Index score), characteristics of participants (number, age, gender), CIMT (mean and SD), FMD (mean and SD), matched factor, whether exclusion of cardiovascular risk factors in participant selection. The methodological quality of the selected studies was assessed by using the Newcastle–Ottawa Scale (NOS)^32^ with the following 3 items: selection of the study groups, between-group comparability, and the ascertainment of either the exposure or the outcome. Study with NOS score ≥5 was judged to be of higher quality.

### Statistical Analyses

CIMT and FMD were expressed as continuous data. The pooled effect size was calculated as the weighted mean difference (WMD) with 95% confidence interval (CI) by the inverse variance optimal approach between the psoriatic patients and control group. The degree of heterogeneity across studies was tested by using the *I*^2^ statistic and Cochran Q statistic. A *I*^2^ statistic value <50% or Cochran Q value of *P* < 0.05 was considered substantial heterogeneity. Random-effects analysis was used to estimate the effect size because of the anticipated clinical heterogeneity among included studies. The presence of publication bias was investigated by the Egger regression^33^ and Begg correlation test^34^ with a *P* value <0.01, which is statistically significant. Subgroups analyses were performed according to the type of disease (psoriasis or psoriatic arthritis), study design (case-control or cross-sectional), matched factor (matched BMI or not), mean age of psoriatic patients (>45 years or <45 years), and whether participants with cardiovascular risk factors were excluded. All statistical analyses were performed with Stata software 12.0 (Statacorp, College Station, TX).

## RESULTS

### Study Selection

**FIGURE 1:**
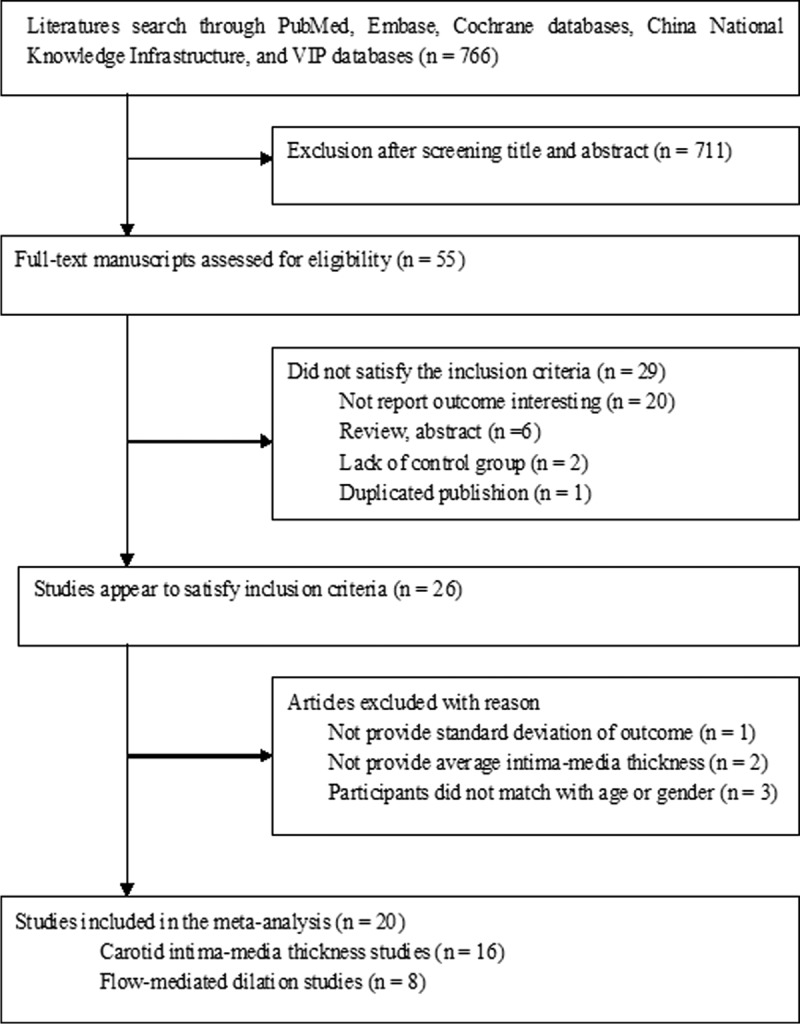
Flowchart of the study selection.

The initial literature search yielded a total of 766 potentially relevant articles. After screening on the basis of abstracts or titles, 711 articles were excluded. After full manuscripts assessed for eligibility, 26 articles appeared to satisfy the inclusion criteria. Six articles were excluded for the following reasons: one study^35^ did not provide SD of the outcome; 2 studies^36,37^ did not provide average intima-media thickness values; and participants’ selection did not match with age or sex in 2 studies.^38,39^ One study^40^ was further excluded because the reported FMD value was particularly high in both psoriatic patients and controls than other studies. Finally, only 20 studies^10–28,30^ were included in the quantitative synthesis. The flowchart of the studies’ selection process is outlined in Figure 1.

### Study Characteristics

Twenty studies^10–28,30^ comprising 1066 psoriatic patients and 962 controls were included in the meta-analysis. The sample size of individual study ranged from 54 to 196. Of the 20 studies, 16^10,11,13–19,21–23,25,27,28,30^ were case–control studies, 3^12,20,24^ were cross-sectional studies, and 1^26^ was cross-sectional plus case–control design. All the studies were published from 2007 to 2014. All studies clearly reported matching of patients and controls by sex and age design. Ten studies excluded participants with preexisting cardiovascular risk factors. The studies enrolled participants with a mean age range from 30 to 57.85 years. The characteristics of the included studies are shown in Table 1 .^10–28,30^ All quality scores of the included studies were >5 according to the NOS. NOS scores of the included studies are presented in Table 2.^10–28,30^

**TABLE 1:**
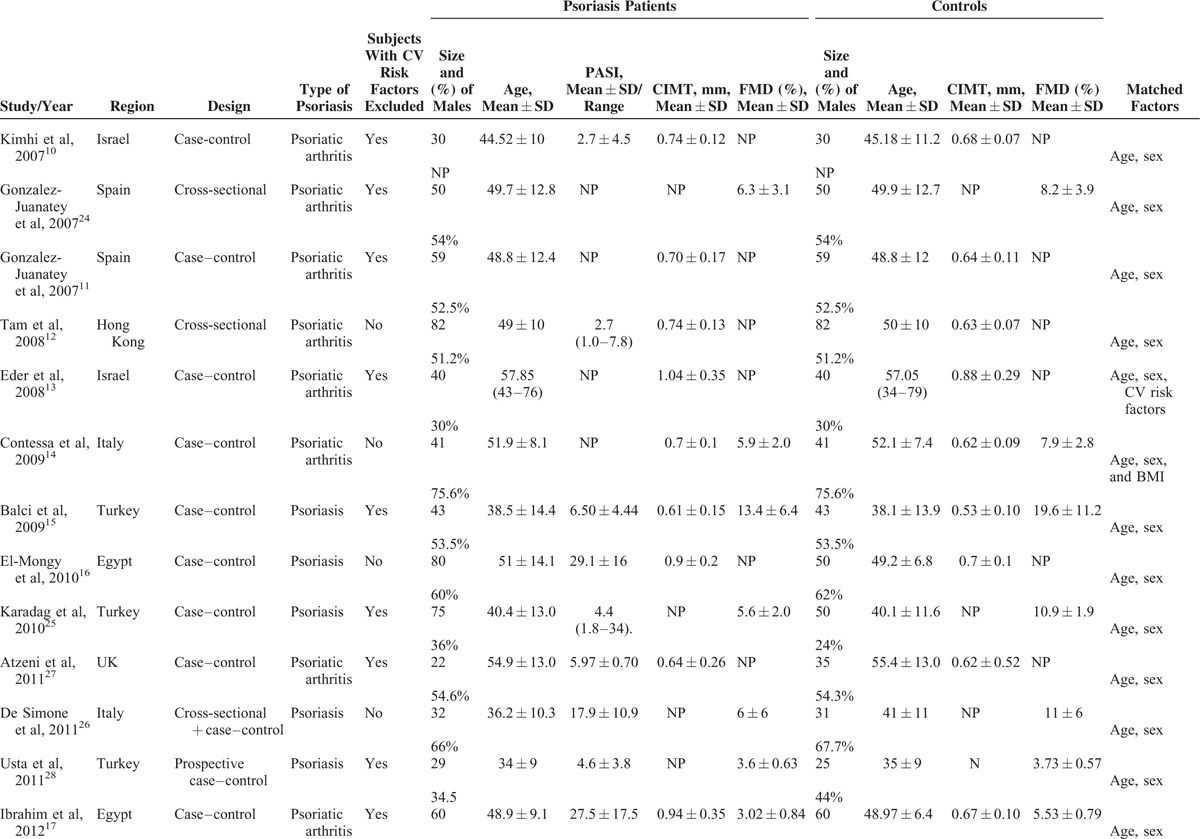
Baseline Characteristics of Studies Included in the Meta-Analysis

**TABLE 1 (Continued):**
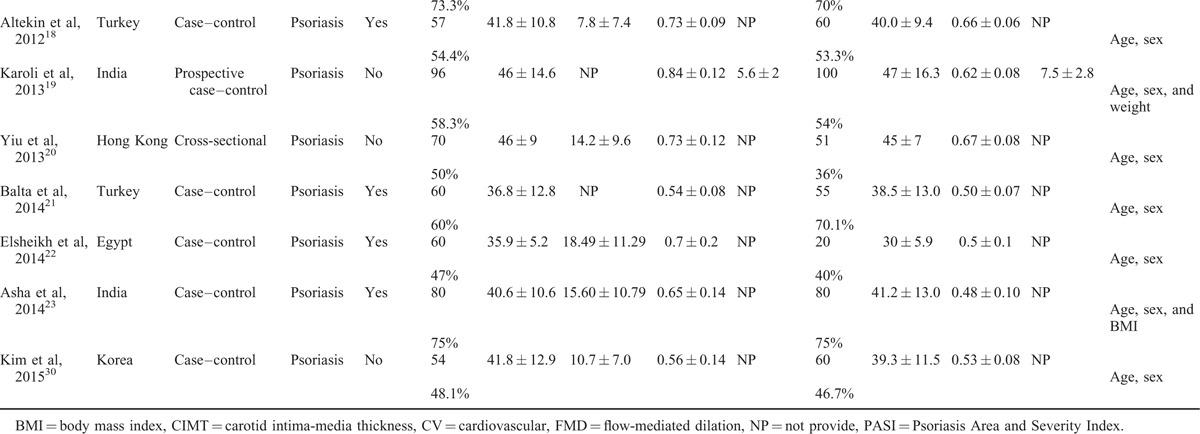
Baseline Characteristics of Studies Included in the Meta-Analysis

**TABLE 2:**
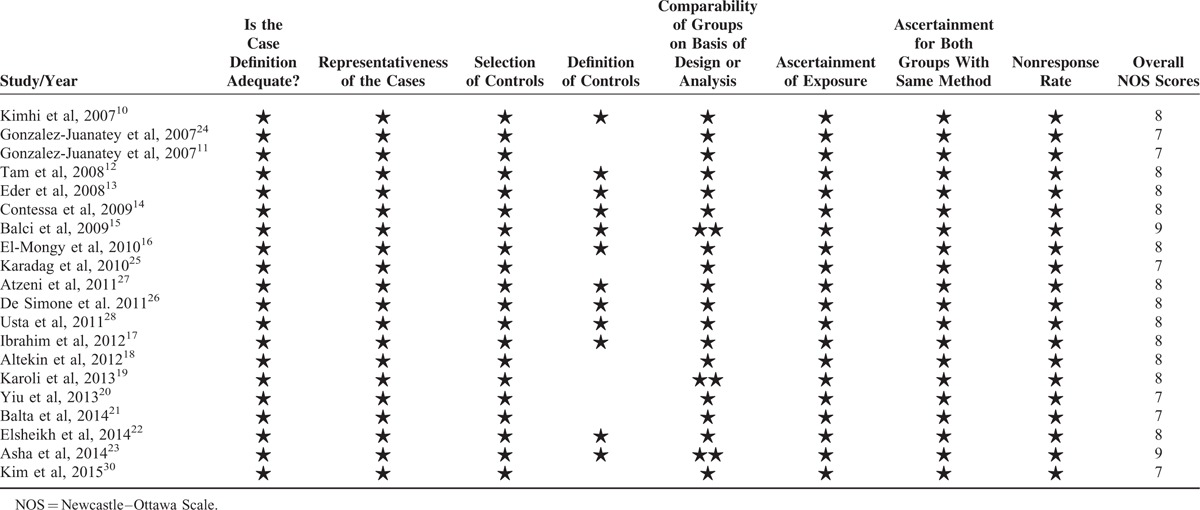
Quality Assessment of Studies Included in Meta-Analysis

### Psoriasis and CIMT

**FIGURE 2:**
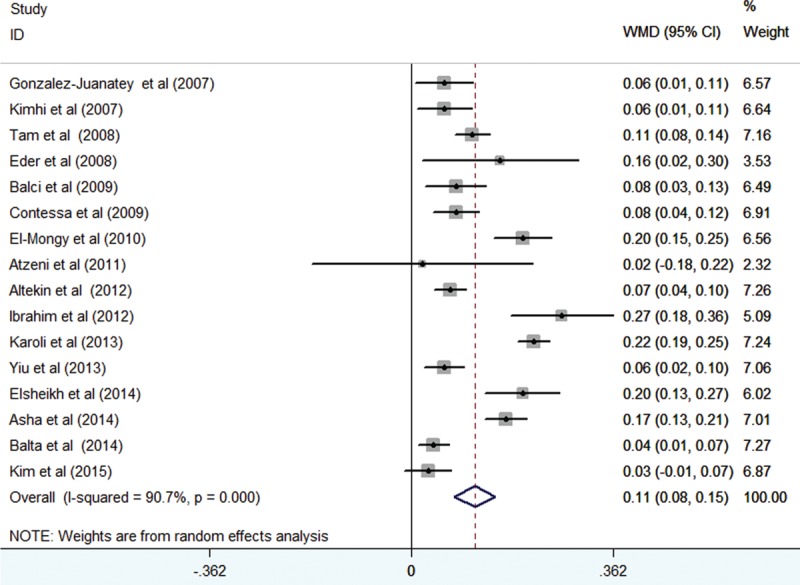
Forest plots showing the weighted mean difference of carotid intima-media thickness between psoriatic patients and controls in a random-effects model.

Sixteen studies^10–23,27,30^ assessed the CIMT difference in 934 psoriatic patients and 866 controls. As shown in Figure 2, the pooled random-effect difference in CIMT indicated that psoriatic patients had a significant increase in CIMT compared with controls (WMD 0.11 mm; 95% CI 0.08–0.15). Substantial heterogeneity was observed (*I*^2^ = 90.7%; *P* < 0.001). Neither the Begg rank correlation test (*P* = 0.137) nor the Egger linear regression test (*P* = 0.634) showed evidence of publication bias.

### Psoriasis and FMD

**FIGURE 3:**
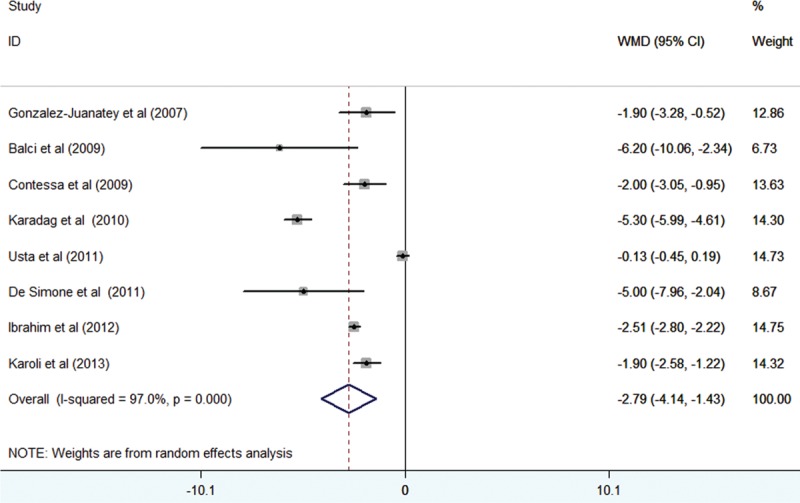
Forest plots showing the weighted mean difference of brachial artery flow-mediated dilation psoriatic patients and controls in a random-effects model.

Eight studies^14,15,17,19,24–26,28^ assessed the FMD difference in 424 psoriatic patients and 400 controls. As shown in Figure 3, the heterogeneity across the included trials was significant (*I*^2^ = 97%; *P* < 0.001), the pooled random-effect difference in FMD showed that psoriatic patients had a significant decrease in FMD than controls (WMD −2.79%; 95% CI −4.14% to −1.43%). Neither the Begg rank correlation test (*P* = 0.348) nor the Egger linear regression test (*P* = 0.149) showed evidence of publication bias.

### Subgroup and Sensitivity Analyses

**FIGURE 4:**
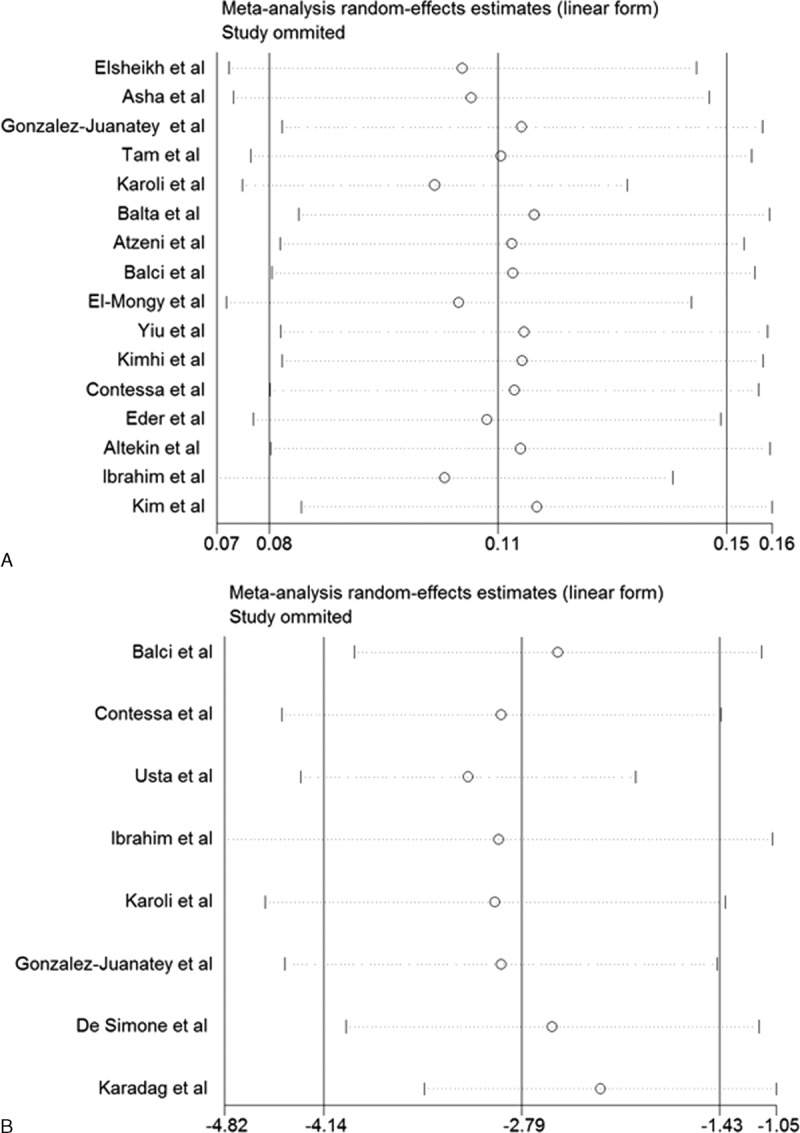
Sensitivity analyses on carotid intima-media thickness (A) and brachial artery flow-mediated dilation (B).

Subgroup analyses showed that psoriatic arthritis alone appeared to have less CIMT (WMD 0.10 mm; 95% CI 0.06–0.15) and less impaired FMD (WMD −2.45%; 95% CI −2.73 to −2.18). Cardiovascular risk factors, age, and BMI of participants could influence the results. The detailed results of the subgroup analyses are shown in Table 3. Sensitivity analyses indicated that the pooled effect sizes of WMD for CIMT (Figure 4A) or FMD (Figure 4B) changed very little by sequential omission of individual trials.

**TABLE 3:**
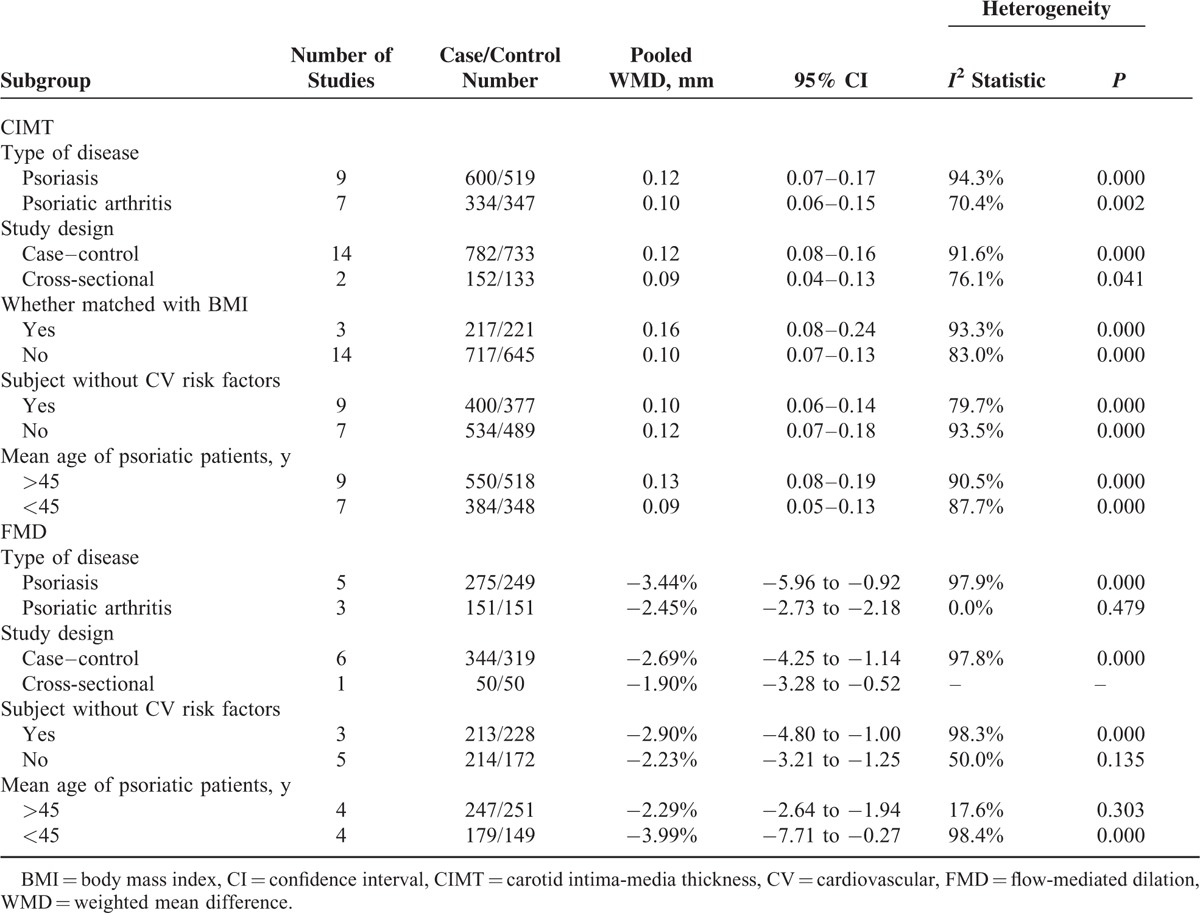
Subgroup Analyses of CIMT and FMD

## DISCUSSION

This meta-analysis suggests that patients with psoriasis are associated with an increased CIMT and impaired brachial artery FMD than the healthy controls, which reflects the subclinical atherosclerosis. Compared with controls, psoriatic patients had 2.79% reduction in brachial artery FMD and 0.11 mm thicker CIMT. Particularly, psoriatic arthritis had less impaired brachial artery FMD and thinner CIMT than total psoriatic patients.

When we investigated the relationship between psoriasis and subclinical atherosclerosis, the impact of type of disease, BMI, concomitant cardiovascular risk factors, and age should be considered. Subgroup analysis showed that patients with psoriatic arthritis appeared to have less impaired brachial artery FMD and thinner CIMT than the total psoriatic patients. CIMT may be affected by other cardiovascular risk factors. However, WMD of impaired FMD or increased CIMT remained statistically significant for participants without clinical evidence of atherosclerotic risk factors. These findings implied that the clustering of cardiovascular risk factors in psoriatic patients may amplify the effect of psoriasis on subclinical atherosclerosis. Psoriatic patients had a higher prevalence and incidence of obesity.^41^ Subgroup analysis indicated that the pooled WMD of CIMT was increased when we pooled studies of matching factor with BMI. Together these findings, psoriatic patients were associated with accelerated subclinical atherosclerosis and might be independent of the classical atherosclerotic risk factors.

Elder people are usually associated with accelerated atherosclerosis. In this study, psoriatic patients with mean age >45 years appeared to have greater thicker CIMT than those younger than 45 years. On the contrary, the impaired brachial artery FMD was more pronounced in patients with mean age <45 years. Impaired endothelial function might precede any change in CIMT. These findings revealed that determination of FMD may be recommended for psoriatic patients with mean age <45 years, whereas measurement of CIMT might be suitable for the older patients.

The association between psoriasis and risk of cardiovascular disease is controversial. A well-designed meta-analysis suggested that psoriasis was associated with ischemic heart disease but not cerebrovascular disease and cardiovascular mortality.^42^ Our meta-analyses indicated that patients with psoriasis were associated with excessive risk of subclinical atherosclerosis. Accordingly, a systematic review summarized that patients with psoriasis and psoriatic arthritis had impaired endothelial function compared with the general population, as measured by pulse wave velocity and aortic stiffness parameters.^43^ A more recent published meta-analysis^44^ suggested that patients with psoriatic arthritis appeared significantly associated with markers of subclinical atherosclerosis. However, this meta-analysis mainly focused on patients with psoriatic arthritis but not address total psoriatic patients. These meta-analyses supported that patients with psoriasis may increase future cardiovascular morbidity and mortality.

The exact mechanisms of psoriasis in promoting atherosclerosis remain unclear. Psoriasis is considered a systemic inflammatory condition. The chronic systemic inflammatory state has been linked to an acceleration of the atherosclerotic lesions. Chronic systemic inflammation induces endothelial dysfunction, altered glucose metabolism, and insulin resistance that play a significant role in the progress of atherosclerosis.^45,46^ Moreover, many immunological factors involved in psoriasis, such as C-reactive protein and tumor necrosis factor-α, also contribute to atherosclerosis.^47^

Several limitations should be considered. First, the causal association between subclinical atherosclerosis and psoriasis could not be defined because of the case–control or cross-sectional nature of the included studies. Second, studies using other techniques to evaluate subclinical atherosclerosis were not included in this meta-analysis. Third, as for the included studies did not provide data about the severity or duration of psoriasis on the subclinical atherosclerosis, so we could not determine whether the severity of psoriasis or longer duration of the disease increased the extent of subclinical atherosclerosis. Fourth, significant heterogeneity in pooled CIMT (*I*^2^ = 90.7%) and FMD (*I*^2^ = 97%) was observed. The sources of heterogeneity might be correlated with the study design, age of the participant, presence or absence of psoriatic arthritis, and with or without atherosclerotic risk factors. Finally, we were unable to determine the effects of pharmacologic therapy on the progression of atherosclerosis in psoriatic patients.

## CONCLUSIONS

This meta-analysis suggests that psoriatic patients are associated with excessive risk of subclinical atherosclerosis compared with the healthy controls. Assessment of CIMT and FMD of the brachial artery may be recommended to identify a subgroup of patients at higher risk for cardiovascular events in psoriatic patients. Psoriatic patients with mean age >45 years appeared to have greater thicker CIMTA and need frequent follow-up. Moreover, further studies are needed on whether the treatment of psoriasis will reverse subclinical atherosclerosis.
